# The complete mitochondrial genome of the assassin bug *Reduvius gregoryi* (Hemiptera: Reduviidae)

**DOI:** 10.1080/23802359.2019.1666690

**Published:** 2019-09-20

**Authors:** Qiaoqiao Liu, Fan Song, Wanzhi Cai, Hu Li

**Affiliations:** Department of Entomology and MOA Key Lab of Pest Monitoring and Green Management College of Pant Protection, China Agricultural University, Beijing, China

**Keywords:** Mitochondrial genome, Hemiptera, Reduviidae, *Reduvius gregoryi*

## Abstract

The complete mitochondrial genome (mitogenome) of the assassin bug, *Reduvius gregoryi*, was determined. The sequenced mitogenome is a typical circular DNA molecule of 16,477 bp, containing 13 protein-coding genes, 2 rRNA genes, 22 tRNA genes and a putative control region. Protein-coding genes all initiate with ATN codons and terminate with TAA codons except for *ATP6*, *COI, COIII, ND4,* and *ND5* use a single T residue as the termination codon. All tRNAs have the clover-leaf structure except for the *tRNA^Ser(AGN)^* and the length of them range from 61 to 70 bp. The control region is 1731 bp long with an A + T content of 72.3%. Our phylogenetic analysis supported the polyphyly of Reduviinae and the sister relationship between *Reduvius gregoryi* and *Reduvius tenebrosus*.

The genus *Reduvius* Fabricius is one of the most speciose genera of assassin bugs (Hemiptera: Reduviidae) worldwide. About 197 species have been recognized in this genus so far (Weirauch et al. 2015). Most species occur in arid- and semi-arid areas in the Afrotropical, Oriental, and Palearctic regions. To date, only one mitogenome have been sequenced from the genus *Reduvius* (Jiang et al. 2016). Here, we sequenced the complete mitogenome of *Reduvius gregoryi*, which is the second representation of *Reduvius*. The samples were collected in Medog country, Xizang Autonomous Region, China (29°39′17″N 95°29′26″E). Voucher specimen was deposited at the Entomological Museum of China Agricultural University (No. VCim-00112) and the sequence was deposited in GenBank under the accession number KY069969.

This sequenced mitogenome is 16,477 bp long, including 37 genes (13 protein-coding genes, 22 tRNA genes, and 2 rRNA genes) and a control region. Gene order is identical to the putative ancestral arrangement of insects and other assassin bugs (Cameron 2014; Song et al. 2016; Li et al. 2017; Song et al. 2019). Except control region, this mitochondrial genome has one 182 bp inter-genic regions, which is between *ND1* and *tRNA^Ser(UCN)^*. There are totally 56 bp overlapped nucleotides between neighboring genes in 14 locations, ranging from 1 to 18 bp in size.

The nucleotide composition of the whole mitogenome is significantly biased toward A + T (71.1%) with positive AT-skew (0.19) and negative GC-skew (−0.26). All protein-coding genes initiate with ATN as the start codon (2 with ATA, 5 with ATT, and 6 with ATG). The stop codon TAA/TAG was assigned to 8 protein-coding genes. *ATP6*, *COI, COIII, ND4,* and *ND5* used a single T residue as incomplete stop codon which is commonly reported in insect mitogenomes (Wang et al. 2014).

The length of the 22 sequenced tRNA genes range from 61 to 70 bp. Among all tRNA genes, only *tRNA^Ser(AGN)^* cannot exhibit the classic cloverleaf secondary structure, due to the deficiency of the dihydrouridine (DHU) arm which is typical feature of insect mitogenomes (Li et al. 2012). The *lrRNA* is 1255 bp long with an A + T content of 72.5% and the *srRNA* is 786 bp long with an A + T content of 71.8%. The control region, which is located between *srRNA* and *tRNA^Ile^*, is 1731 bp long and is also significantly biased toward A + T (72.3%).

We analyzed nucleotide sequences of 13 protein-coding genes and 2 rRNAs with maximum likelihood (ML) method to understand the phylogenetic relationships within Reduviidae (Figure 1). The two *Reduvius* species were clustered into a branch with 100 bootstrap values. The subfamily Reduviinae was polyphyletic which was also recovered in previous comprehensive taxa-sampling studies (Weirauch 2008; Hwang and Weirauch 2012; Liu et al. 2018).

**Figure 1.**
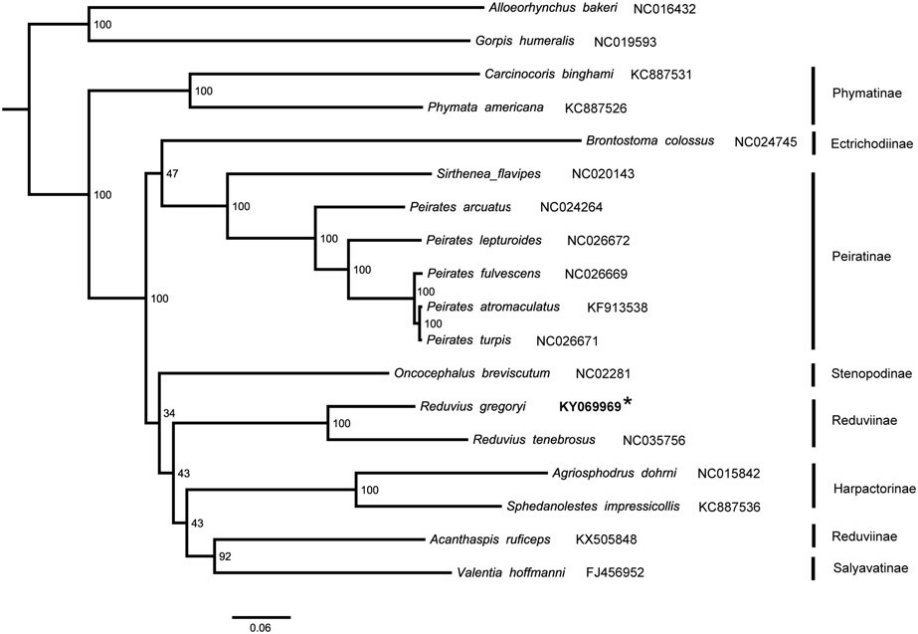
Maximum-likelihood (ML) phylogenetic tree of 16 Reduviidae species inferred from analysis of the 13 protein-coding genes and 2 rRNAs genes (12,697 bp) and generated by IQ-TREE 1.6.5 (Trifinopoulos et al. 2016). Number above each node indicates the ML bootstrap support values. Alphanumeric terms indicate the GenBank accession numbers.
